# Point-of-care HPV testing for cervical cancer screening in Sub-Saharan Africa: platform diversity, diagnostic performance, implementation feasibility, and determinants—a scoping review with contextual considerations for Ethiopia

**DOI:** 10.1186/s12889-026-26382-9

**Published:** 2026-01-26

**Authors:** Melkamu Siferih, Getnet Gedif, Anteneh Lamesgen, Alehegn Aderaw Alamneh, Abebaw Abeje Muluneh, Yilkal Dagnaw Melesse, Getachew Tilaye Mihiret, Menberu Gete, Asmamaw Getnet, Haile Amha, Aysheshim Asnake Abneh, Atsede Alle Ewunetie, Tadele Derbew Kassie

**Affiliations:** 1 Department of Obstetrics and Gynecology, School of Medicine, College of Medicine and Health Sciences, Debre Markos University, Debre Markos, Ethiopia; 2 Department of Public Health, College of Medicine and Health Sciences, Debre Markos University, Debre Markos, Ethiopia; 3 Department of Human Nutrition, College of Medicine and Health Sciences, Debre Markos University, Debre Markos, Ethiopia; 4 Department of Midwifery, College of Medicine and Health Sciences, Debre Markos University, Debre Markos, Ethiopia; 5 Department of Nursing, College of Medicine and Health Sciences, Debre Markos University, Debre Markos, Ethiopia

**Keywords:** Point-of-care HPV, Cervical cancer, Sub-Saharan Africa, Diagnostic performance, Implementation feasibility, Ethiopia, Low-resource-settings

## Abstract

**Background:**

Cervical cancer remains a leading cause of cancer-related mortality among women in Sub-Saharan Africa (SSA), where access to laboratory-based screening is limited. Point-of-care (POC) HPV testing enables rapid, near-patient diagnostics that support same-day screen- and treat strategies; however, the evidence base across SSA remains fragmented.

**Methods:**

We conducted a scoping review following the Arksey and O’ Malley and the Joanna Briggs Institute (JBI) framework. Ten electronic databases and grey literature sources (2000–2025) were searched for studies evaluating POC HPV tests in SSA. Data on platform characteristics, diagnostic performance, feasibility, acceptability, barriers, facilitators, and sustainability were extracted and synthesized.

**Results:**

Thirty-one unique studies from 15 SSA countries evaluated seven POC HPV platforms (Xpert HPV, careHPV™, AmpFire HPV, LAMP (DARQ), ScreenFire, 8-HPV OncoE6/E7, OncoE6™). Xpert HPV consistently demonstrated high sensitivity for CIN2 + lesions (71–97%) and CIN3 + lesions (68–99%) based on histology-confirmed lesions, with moderate specificity (59–71%) when using biopsy-confirmed histopathology as the reference standard. OncoE6™ exhibited high specificity (98–99%) but lower sensitivity (31–57%). CareHPV performance varied widely (sensitivity 54–97%, specificity 54–94%) and, as with other POC HPV platforms, was influenced by HIV status, sample type, and clinical setting. Acceptability among women was high, especially for self-sampling and rapid results; provider acceptability was supported by minimal training requirement. Evidences from implementation studies suggest that POC HPV testing is operationally feasible in SSA, though key barriers include test costs, supply chain interruptions, and challenges in patient follow-up. Facilitators for feasibility and scale-up include integration with HIV/TB services, community-based outreach, and streamlined workflows. However, data on large-scale implementation, cost-effectiveness, and sustainability remain limited.

**Conclusion:**

POC HPV testing in SSA demonstrates adequate diagnostic performance and high acceptability, and feasibility under programmatic conditions. In settings with healthcare infrastructure similar to that of Ethiopia, successful scale-up of POC HPV testing will require integration with the existing health services, strengthened quality assurance, providers training, and robust patient follow-up mechanisms. Future research should focus on implementation trials, cost-effectiveness, effective triage strategies, and long-term sustainability.

**Supplementary Information:**

The online version contains supplementary material available at 10.1186/s12889-026-26382-9.

## Introduction

Cervical cancer remains the leading cause of cancer morbidity and mortality among women in Sub-Saharan Africa (SSA), accounting for a disproportionate share of global disease burden [1–3]. In response, the World Health Organization (WHO) has set ambitious “90-70-90” targets to eliminate cervical cancer as a public health problem by 2030: 90% of girls fully vaccinated against HPV, 70% of women screened using a high-performance test by age 35 and again by 45, and 90% of women with precancer or cancer receiving appropriate treatment. While these targets are challenging globally, achieving them remains particularly difficult in low- and middle-income countries (LMICs) due to limitations in infrastructure, resources, and access to care, although high-income countries also face implementation challenges [4–8].

Human papilloma virus (HPV) testing is now recognized as the most sensitive method for the early detection of cervical precancerous lesions, enabling timely intervention, and reducing cervical cancer incidence [9, 10]. Yet, conventional laboratory-based molecular testing are largely inaccessible in SSA due to limited laboratory infrastructure, logistical barriers, and prolonged turnaround times [11–13]. These constraints leave many women unscreened or untreated, perpetuating high mortality rates [13, 14].

Point-of-care (POC) HPV testing platforms, such as Xpert HPV, careHPV, AmpFire, 8-HPV OncoE6/E7, OncoE6^™^, and other newer technologies offer rapid, near- patient diagnostics and the potential for same-day screen-and-treat strategies [15–17]. These technologies hold promise for transforming cervical cancer prevention programs in SSA, by increasing coverage, minimizing loss-to-follow-up, and supporting decentralized care models [18, 19]. However, diagnostic performance, feasibility, and implementation determinants vary across countries, healthcare settings, populations, underscoring the need for context-specific evidence [13, 19].

Despite growing interest, no comprehensive synthesis currently maps the diagnostic accuracy, operational feasibility, and implementation determinants of POC HPV testing across SSA.This gap is particularly critical for Ethiopia, a high-burden low-coverage setting where cervical cancer is the second most common cancer among women and population-level screening coverage remains below 10% [20–23]. Understanding the regional evidence and its applicability to Ethiopia is essential to inform national policy, optimize resource allocation, and accelerate progress to cervical cancer elimination [4, 5, 24, 25]. In Ethiopia, laboratory-based HPV testing capacity is extremely limited outside major urban centers, and current screening efforts rely predominantly on visual inspection with acetic acid (VIA), which faces well-documented challenges related to sensitivity, quality assurance, and loss to follow-up. In this context, POC HPV testing offers a potentially transformative opportunity to enable high-performance screening, decentralized services, and support same-day screen-and-treat models [26–30]. However, despite this relevance, locally generated evidence on POC testing in Ethiopia remains scarce, necessitating cautious interpretation of regional data when drawing policy-relevant lessons.

This scoping review therefore aims to synthesize existing current evidence on POC HPV testing, with particular attention to diagnostic performance, acceptability, feasibility, and contextual determinants, while critically examining the applicability and limitations of this evidence for informing cervical cancer screening strategies in Ethiopia.

## Objectives

### General objective

To comprehensively map and synthesize evidence on POC HPV testing for cervical cancer screening in Sub-Saharan Africa, a focus on platform diversity, diagnostic performance, implementation feasibility, and contextual determinants, to inform evidence-based cervical cancer screening strategies in Ethiopia.

### Specific objectives

1. Identify POC HPV testing platforms and summarize their diagnostic performance in SSA
2. Examine implementation outcomes (acceptability, feasibility, uptake, workflow integration, and sustainability)
3. Synthesize contextual barriers and facilitators shaping POC HPV testing across SSA
4. Interpret cross-country evidence to draw considerations relevant for Ethiopia

### Research questions

1. Which POC HPV platforms are implemented in SSA and what evidence exists on their diagnostic accuracy
2. What are implementation outcomes (acceptability, feasibility, uptake, workflow integration, and sustainability)?
3. What contextual determinants affect success or failure of POC HPV deployment?
4. What considerations can be drawn from SSA to guide the scale-up of POC HPV testing?

## Methods

### Methodological framework

This scoping review followed the Arksey and O’Malley (2005) framework, refined by Levac et al. (2010) and the Joanna Briggs Institute (JBI) scoping review manual (2020). Reporting aligns with the PRISMA-SCR (Preferred Reporting Items for Systematic Reviews and Meta-analyses extension for Scoping Reviews) checklist.

### Eligibility criteria (PCC Framework)

Eligibility was defined using population—concept—context (PCC) Framework (Table 1).

**Table 1:** Eligibility criteria for inclusion of studies (PCC Framework)

Criteria	Description
Population	Women eligible for cervical cancer screening in SSA (any adult age, including WLWHIV) and healthcare providers involved in POC HPV testing in SSA. Studies were included regardless of age variation, provided participants met local screening eligibility.
Concept	Evaluation of POC HPV testing (HPV DNA) for diagnostic accuracy, diagnostic performance, implementation outcomes (acceptability, feasibility, uptake, workflow integration, and sustainability), and contextual determinants
Context	Facility-bases, outreach, or community-based settings in any SSA country

### Inclusion criteria

1. Original studies assessing POC HPV tests, including diagnostic accuracy, feasibility, or implementation studies
2. Quantitative, qualitative, and mixed-method studies
3. Grey-literature (reports, theses, conference papers) with sufficient methodological details
4. Publications from 2000 onward, in English

### Exclusion criteria

1. Studies outside SSA
2. Laboratory-based HPV testing without a point of care component
3. Reviews, editorials, and commentaries without primary data
4. Non-cervico-vaginal samples

### Information sources

A comprehensive search was conducted in PubMed/MEDLINE, Scopus, Web of Science, Ovid/Medline, Cochrane library, ScienceDirect, African Index Medicus, and African Journals Online. Grey literature sources included Google Scholar and ProQuest. Searches were restricted to English-language publications from 2000 onward and conducted between October 1–15, 2025. Search strategies were tailored to each database to ensure maximal retrieval.

To enhance comprehensiveness, the reference lists of all included studies were systematically screened to identify additional potential eligible articles. Any studies identified through this manual search were evaluated against the same predefined eligibility criteria applied to database-identified records.

### Search strategy

The search combined seven key concepts: point-of-care-testing, HPV, diagnostic/screening, disease focus, performance, implementation outcomes, and geographical scope. A summary of MeSH terms and keywords is provided in Table 2. The full PubMed search strategy is provided in Appendix 1. Equivalent strategies were adapted for all other databases.

**Table 2:** Summary of search concepts and terms

Concept	MeSH Terms/ Keywords
Point-of-care-testing	“Point-of-care-systems’’ [MeSH], point-of-care, rapid, near-patient, same-day, onsite, bedside
HPV	“Papilloma virus infection’’[MeSH], HPV, Human papilloma virus, HPV DNA, HPV testing, HPV assay
Diagnostic/screening	“Molecular diagnostic techniques’’[MeSH], testing, screening, diagnostic, diagnosis, detection, nucleic acid test
Disease focus	“Uterine Cervical Neoplasms’’[MeSH], cervical cancer, cervical neoplasia, cervical lesions, CIN, cervical screening
Geographical scope	“Africa South of the Sahara’’[MeSH], Sub-Saharan Africa, SSA, Ethiopia, Kenya, Uganda, Tanzania, Nigeria, Ghana, Zambia, Malawi, Zimbabwe, Rwanda, Burundi, South Africa
Performance	“Sensitivity and Specificity’’[MeSH], Accuracy, sensitivity, specificity, predictive value, diagnostic performance, validity, reliability
Implementation outcomes	“Patient acceptance of Healthcare’’[MeSH], “Feasibility Studies’’[MeSH], “Implementation Sciences’’[MeSH], “Health Service accessibility’’[MeSH], feasibility, acceptability, implementation, uptake, barriers, facilitators, determinants, scalability, sustainability, cost-effectiveness

### Study selection

All identified records were imported into EndNote for deduplication. Study selection followed a two stage process in accordance with PRISMA-SCR guidelines. First, MS and TD independently screened titles and abstracts against predefined eligibility criteria; discrepancies were resolved through discussion. Second, full-text articles were independently reviewed, with disagreements resolved by consensus.

### Data extraction

Data were charted using a predefined Excel template aligned with PRISMA-SCR. Two reviewers independently extracted:

• Study characteristics (author, year, country, setting, study design, population, sample size)
• POC platform details (test type, HPV types detected, HPV positivity, specimen type, self- vs. provider-collected)
• Participant factors (age, HIV status, uptake)
• Diagnostic accuracy: sensitivity, specificity, PPV, and NPV with 95% confidence interval, reference standards
• Implementation elements: screening model (same-day, sequential, or community outreach), women’s and provider’s acceptability, and feasibility indicators (infrastructure needs, workflow, equipment, suitability for HIV clinics)
• Barriers and facilitators: stock outs, test cost, power interruptions, training gaps, staff turnover, linkage-to-treatment outcomes, and sustainability/scalability notes
• Authors’ conclusions

### Data synthesis

A combined descriptive and thematic approach was employed. Quantitative outcomes (uptake, positivity, diagnostic accuracy) were summarized using descriptive statistics and presented in structured tables. Qualitative and implementation- related findings were synthesized narratively and categorized into key themes: acceptability, feasibility, barriers, facilitators, and sustainability. Evidence mapping characterized the scope of POC HPV testing research and highlighted knowledge gaps and identified implementation challenges. Meta-analysis was not performed due to heterogeneity of study designs and outcomes.

## Results

**Fig. 1:**
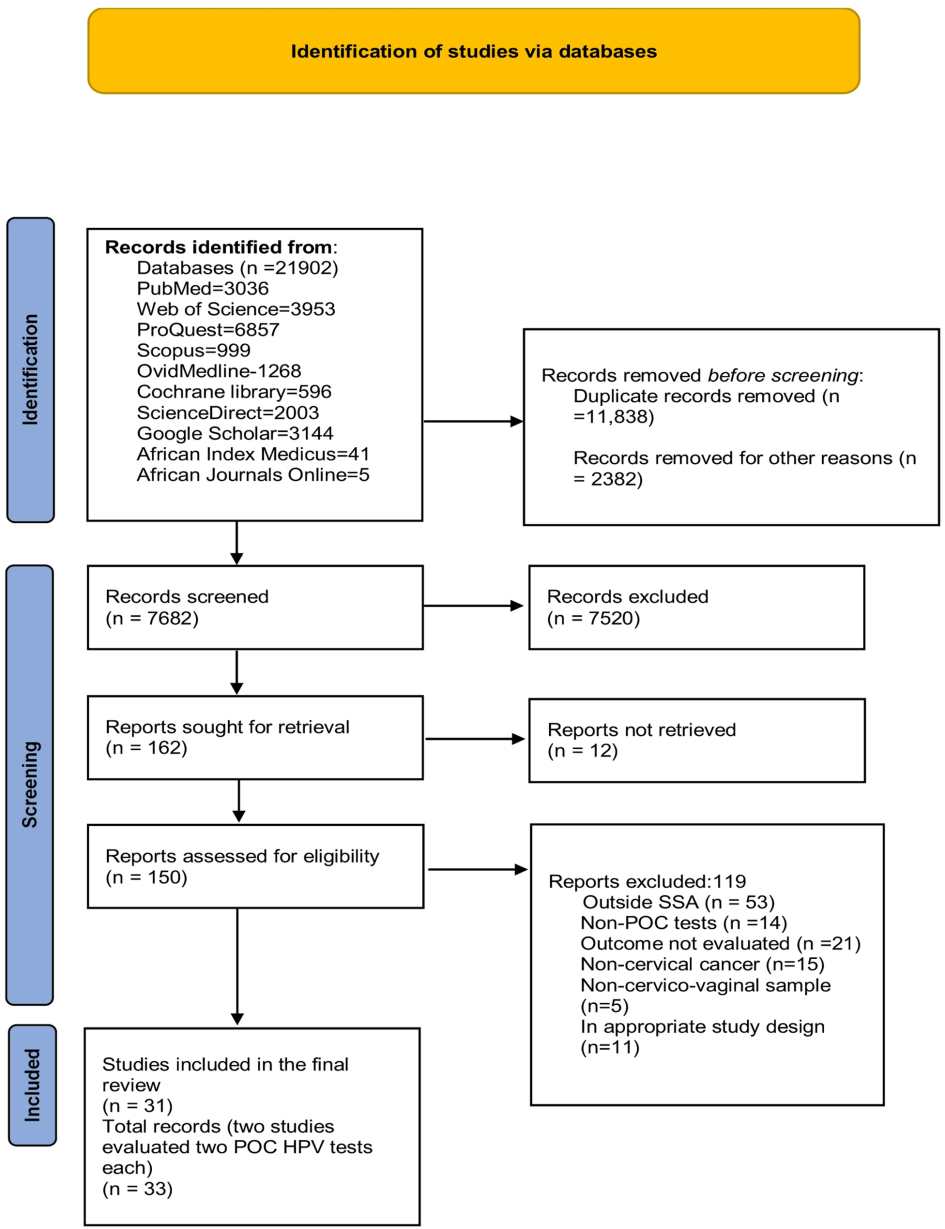
PRISMA-SCR flow diagrams

A total of 21,902 records were identified through ten databases. Following removal of 11,838 duplicates and 2,382 administrative or technical records, 7682 unique citations were screened by title/abstract. Of these, 7520 records failed to meet inclusion criteria. We retrieved 162 full-text articles; 12 could not be obtained despite repeated attempts. Of the 150 full-text articles assessed, 119 articles were excluded for the following reasons: study conducted outside sub-Saharan Africa (*n* = 53), non-POC HPV test (*n* = 14), irrelevant outcomes (*n* = 21), non-cervical cancer focus (*n* = 15), ineligible specimen type (*n* = 5), or ineligible study design (*n* = 11). Ultimately, 31 unique studies met all inclusion criteria and were synthesized. Two of these studies evaluated two POC HPV platforms each; therefore, their findings are presented separately for each platform, resulting in 33 records in total to allow detailed descriptions for each. Across these studies 42 POC HPV tests were identified because several studies assessed multiple platforms or testing arms. These studies constitute the current evidence base for POC HPV testing for cervical cancer screening in sub-Saharan Africa. A PRISMA-SCR flow diagram summarizes the selection process (Fig. 1).

### Characteristics of included studies

The included studies spanned across 15 Sub-Saharan African countries with the largest contributions from South Africa (8 studies), Tanzania (4), Ghana (3), Malawi (3), Zambia (3), Botswana (3), and smaller but important representations from East, West, and Central Africa. Notably, Ethiopia contributed only one eligible study to the evidence base, highlighting the scarcity of locally generated evidence on POC HPV testing and the need for further primary research to inform the national cervical screening strategies. Most studies were conducted in clinic-based screening programs (≈ 28), complemented by community outreach initiatives (≈ 6) and laboratory-based validations or analytical studies (≈ 6), reflecting diverse healthcare delivery contexts.

Study designs included cross-sectional, prospective observational, diagnostic accuracy evaluations, comparative assays, validation studies, and implementation/pilot studies, consistent with the scope of implementation science, program evaluation, and diagnostic accuracy. Sample sizes varied widely (63 − 16,951 participants), supporting both small scale analytical comparisons and large population-based mass-screening programs. Women aged ˜18–65 years were included, spanning both HIV-positive and HIV-negative populations, an important consideration given the known interaction between HIV infections and Human Papillomavirus (HPV) persistence. Detailed POC testing platforms, study designs, and participant characteristics in Sub-Saharan Africa are provided in Supplementary Table S1.

Across the included studies, seven primary POC platforms were evaluated—Xpert HPV (Cepheid GeneXpert), careHPV (QIAGEN), AmpFire HP, 8-HPV OncoE6/E7, OncoE6™ (Arbor Vita)), LAMP (DARQ) and ScreenFire), with several studies conducting direct comparisons or analytical validations. Xpert HPV and careHPV dominated evaluations due to WHO prequalification and their established presence in LMICs diagnostic networks, while newer assays (AmpFire HPV, LAMP, ScreenFire) broadened the landscape of ultra-rapid, decentralized HPV testing, and Xpert HPV was the most extensively assessed platform overall. POC platforms exhibited considerable variability in diagnostic performance, with Xpert HPV demonstrating high sensitivity for CIN2 + lesions (71–97%) and CIN3 + lesions (68–99%) based on histology-confirmed lesions, with moderate specificity (59–71%) when using biopsy-confirmed histopathology as the reference standard. Studies consistently reported rapid turnaround times (< 1 h) and ease of use. Lower sensitivity among women living with HIV was noted in several evaluations. OncoE6™ oncoprotein tests demonstrated very high specificity (98–99%) but lower sensitivity (31–57%), with studies applying these assays primarily for triage purposes. CareHPV performance showed wide variability, with sensitivity ranging from 54% to 97% and specificity 55% to 94% across clinical and outreach settings. As with other POC HPV platforms, variation was associated with differences in sampling method, HIV status, and operational conditions such as batching requirements. Emerging platforms demonstrated encouraging performance. AmpFire HPV showed > 90% concordance with Xpert HPV and rapid sample-to-result time (˜1 h). LAMP (DARQ) and ScreenFire assays reported promising sensitivity and operational feasibility. LAMP’s extraction-free process reduces hands-on time, while ScreenFire provided low-cost risk-based genotyping. Overall, included studies indicated that POC HPV assays achieved performance levels compatible with their intended use in screening and triage pathways (Supplementary Table S2).

Implementation outcomes were reported across clinic-based, outreach, and community-settings. Acceptability was high in all studies assessing user experience; self-sampling acceptance commonly exceeded 80–95%. Rapid turnaround times were associated with high-turn-of results rates. Providers acceptability was linked to minimal training requirements and integration with existing diagnostic systems. Feasibility was demonstrated across diverse service delivery models. Cartridge-based isothermal platforms (e.g. Xpert HPV) showed straightforward workflow integration, whereas batch-based assays (careHPV assays) required additional staffing and coordination. Low electricity assays such as LAMP and ScreenFire were reported as feasible in rural or decentralized facilities. The 8-HPV OncoE6/E7 test, evaluated in the sole implementation trial and the only true POC platform capable of detecting high-risk HPV types 16,18, 45,31, 33, 35, 52, and 58, showed high usability, minimal training needs, and operational feasibility. The most frequently reported barriers included high test cost, limited human resources, stock-outs and supply chain interruptions, need for test batching, inconsistent electricity supply, and limited follow-up mechanisms. Common facilitators included integration with HIV/TB GeneXpert networks, acceptability of self-sampling, community health worker involvement, and digital follow-up tools. Sustainability finding indicated that long-term implementation was more feasible in settings that leveraged existing laboratory or POC diagnostic infrastructure. However, studies highlighted ongoing requirements, stable supply chains, quality assurance systems, and digital data platforms. Detailed implementation models, acceptability, feasibility, barriers, facilitators, and sustainability of POC HPV tests in Sub-Saharan Africa are provided in Supplementary Table S3.

## Discussion

This scoping review comprehensively synthesizes evidence on POC HPV testing for cervical cancer screening across Sub-Saharan Africa, highlighting platform diversity, diagnostic performance, implementation feasibility, and context-specific determinants. The 33 records (derived from 31 unique studies, with two studies evaluating two POC HPV platforms each) demonstrate a rapidly evolving landscape of POC HPV technologies, with Xpert HPV and careHPV dominating the current evidence base, complemented by emerging assays such as AmpFire HPV, LAMP, ScreenFire, 8-HPV OncoE6/E7, and OncoE6™. This diversity reflects ongoing efforts to optimize diagnostic accuracy and workflow efficiency, and to enhance applicability in low-resource decentralized settings.

Globally, POC HPV testing has been prioritized as part of cervical cancer elimination strategies, particularly in low-and middle-income countries (LMICs), where cytology- or VIA-based screening is limited by infrastructure, human resources, and follow-up challenges [31–33]. Our review demonstrates that SSA has actively adopted this approach, although coverage remains uneven, with countries like Ethiopia underrepresented. This highlights the critical need for context-specific evidence to guide national program design.

### Diagnostic performance of POC HPV tests

Xpert HPV consistently demonstrated high sensitivity for CIN2 + lesions (71–97%) and CIN3 + lesions (68–99%) based on histology-confirmed lesions, with moderate specificity (59–71%) when using biopsy-confirmed histopathology as the reference standard, supporting its suitability for primary screening. These findings align with a global meta-analysis reporting pooled sensitivities of 91.5% for self-collected vaginal samples and 92.3% for physician-collected cervical samples, albeit with moderate specificity (56.5% and 53.3%, respectively) [34]. Comparable performance has also been documented across multiple independent studies [35–37]. In contrast, OncoE6™, exhibits exceptional specificity (98–99%) but lower sensitivity (31–57%), consistent with its intended role as a triage assay rather than as a standalone screening test [34, 37, 38]. CareHPV shows variable performance (sensitivity 54–97%; specificity 55–94%), reflecting heterogeneity in sample collection, HIV prevalence, and operational context — a pattern also observed in other POC HPV platforms globally [34, 39, 40].

Emerging platforms such as AmpFire HPV, LAMP, and ScreenFire demonstrated high concordance with Xpert HPV and operational feasibility in SSA. AmpFire HPV reported > 90% agreement with Xpert HPV and rapid turnaround time (˜1 h) [41–43]. LAMP and ScreenFire, with minimal extraction steps and low electricity requirement, further expand options for rural or low-resource settings. These data indicate that both established and emerging POC assays can reliably detect high-risk HPV infections and are compatible same-day screen-and-treat strategies, crucial to minimize loss to follow-up in LMIC contexts [44–46].

### Implementation outcomes: acceptability, feasibility, and operational considerations

Although diagnostic or validation studies dominate the evidence, implementation–focused studies consistently report high acceptability among women, particularly with self-sampling and rapid result delivery (acceptance often > 80–90%) [47–50]. Provider acceptability is bolstered by minimal training requirements, integration with existing HIV/TB or laboratory infrastructure, and simplified workflows [33, 51, 52].

Operational feasibility varied by assay type. Cartridge-based tests such as Xpert HPV, integrated smoothly into routine workflows, whereas batch-dependent assays, including careHPV™, required additional coordination and staffing [17, 53]. Low-resource platforms such as LAMP and ScreenFire were particularly suitable for rural facilities [54–56]. Common barriers included high test costs, stock-outs, supply chain interruptions, batching requirements, and limited follow-up mechanisms [57–61], consistent with experiences in Latin America and Southeast Asia [62]. Facilitators encompassed integration with existing diagnostic networks, self-sampling strategies, engagement of community health workers, and digital follow-up tools [63–66]. Sustainability was greatest in settings leveraging pre-existing diagnostic infrastructure, although long-term scale up requires robust supply chains, quality assurance, and digital tracking systems [59, 67–69].

Downham et al. (2024) conducted the first randomized controlled trial of OncoE6/E7 (8-HPV) assay in Sub-Saharan Africa demonstrating high acceptability among women and providers in both HIV positive and HIV negative populations. While operationally feasible in low-resource laboratories, the assay required batch-based workflow optimization and targeted training. These findings reinforce the potential of OncoE6/E7 to support effective screen-triage-treat strategies and offer valuable insights for scaling novel HPV POC technologies in high-burden settings. Similar experiences have been documented in the literature ( [70, 71].

### Contextual considerations and cautious interpretation for Ethiopia

Although this review highlights important lessons for Ethiopia, it is essential to acknowledge that the Ethiopian-specific evidence base for POC HPV testing is currently very limited. Only one primary study conducted in Ethiopia met the inclusion criteria, focusing on the OncoE6™ Cervical Test. As a result, considerations for Ethiopia are necessarily drawn from evidence generated in other Sub-Saharan African countries with varying health system capacities, diagnostic infrastructure, and implementation contexts. Consequently, recommendations for Ethiopia should be interpreted as context-informed hypotheses rather than definitive conclusions, underscoring the need for local validation and implementation research.

Consistent with this limitation, the review shows that POC HPV testing research in SSA is concentrated in countries such as South Africa, Tanzania, Zambia, and Ghana, while Ethiopia and low-and middle-income countries are underrepresented. This distribution reflects broader global disparities in infrastructure and research investment. Nevertheless, emerging POC platforms with simplified workflows, and minimal resource requirements present opportunities for countries with limited laboratory capacity to adopt decentralized HPV-based screening aligned with global elimination goals [5, 31, 34, 72].

Drawing cautiously from regional evidence, several considerations may inform the phased introduction of POC HPV testing in Ethiopia. Platform selection should account for limited molecular diagnostic infrastructure outside urban centers, favouring assays with simplified workflows, minimal power requirements, and compatibility with existing diagnostic platforms such as GeneXpert systems used in HIV and TB programs [15, 73–75]. Successful implementation will also depend on strengthening health workforce capacity through targeted training of mid-level providers in sample collection, test operation, result interpretation, and referral pathways. Integrating HPV testing into existing reproductive health, HIV, and primary health services may enhance efficiency and coverage, although workflow adaptations and referral mechanisms will require local piloting [76, 77]. Long-term sustainability will depend on reliable procurement systems, uninterrupted supply chains, quality assurance mechanisms, and digital tools for result tracking and patient follow-up. Given the heterogeneity of service delivery contexts, Ethiopia will require locally conducted implementation trials and cost-effectiveness analyses before national scale-up [59, 67–69].

### Research gaps and future directions

Despite promising evidence, key gaps remain. Implementation, cost-effectiveness and long-term outcome studies are sparse in SSA and globally. Head-to-head comparisons of emerging POC platforms are limited, constraining evidence-based decisions for national programs [31, 78–81]. Optimal triage strategies—balancing sensitivity, specificity, and resource use—require further evaluation, particularly in high-risk groups such as women living with HIV [27, 67, 82]. Long-term sustainability, digital health integration, and quality assurance are underreported. Future research in Ethiopia and comparable LMICs should prioritize pragmatic implementation trials, cost-effectiveness analysis, and evaluation of hybrid screening models combining community-based collection with centralized diagnostic hubs [83–86].

### Strengths and limitations of the review

This review provides one of the most comprehensive mappings of POC HPV testing evidence in SSA, integrating diagnostic accuracy, operational feasibility, acceptability, and other implementation outcomes. Strengths include rigorous search strategy, broad inclusion of both established and emerging technologies, and contextualization with global evidence base. However, this review is limited by heterogeneity in study designs, outcome definitions, sampling approaches, reporting metrics, which precluded pooled analyses, and direct quantitative comparisons. In addition, key implementation dimensions—particularly cost, infrastructure requirements, and long-term sustainability—were inconsistently reported, reflecting broader challenges in standardizing evaluations of POC HPV testing.

A further limitation is the limited availability of Ethiopia-specific primary data, with one eligible study identified. Consequently, conclusions related to Ethiopia rely largely on extrapolation from evidence generated in other sub-Saharan Africa Countries, which may differ in health system organization, diagnostic infrastructure, and implementation capacity. While this regional evidence provides valuable insights, it highlights the urgent need for locally generated data to validate feasibility, cost-effectiveness, and sustainability of POC HPV testing within the Ethiopian context.

## Conclusion

Point-of-care (POC) HPV testing has the potential to significantly transform cervical cancer screening in Sub-Saharan Africa across clinic and community settings. WHO-prequalified platforms, Xpert HPV and careHPV provide high sensitivity and acceptable specificity, while emerging assays—AmpFire HPV, LAMP, ScreenFire, OncoE6/E7 (8-HPV), and OncoE6™ —feasible options for decentralized, resource-limited settings. Strategies such as self-sampling, rapid result turnaround, integration with existing health services, and adherence to recommended screening intervals collectively enhance feasibility, acceptability, and population-level coverage. Effective scale-up in Ethiopia and similar contexts will require careful platform selection, robust quality assurance, reliable supply chains, trained personnel, and strengthened patient follow-up and linkage to care. Future research should prioritize pragmatic implementation trials, cost-effectiveness analyses, optimized triage strategies—particularly for high-risk groups, including women living with HIV—and assessment of long-term sustainability. Strengthened evidence on emerging platforms and community-based screening models will be crucial to inform national policy, expand screening coverage, reduce cervical cancer morbidity, and accelerate progress toward global elimination targets.

## Supplementary Information

Supplementary Material 1.

Supplementary Material 2.

Supplementary Material 3.

## Data Availability

All data are included in the article and supplementary files.

## Abbreviations

CIN2+: Cervical Intraepithelial Neoplasia Grade 2 or higher
CIN3+: Cervical Intraepithelial Neoplasia Grade 3 or higher
HPV: Human Papilloma Virus
JBI: Joanna Briggs Institute
LAMP: Loop-mediated Isothermal Amplification
LMICS: Low-and Middle Income Countries
NPV: Negative Predictive Value
POC: Point-of-Care
SSA: Sub-Saharan Africa
WLIHIV/WLWH: Women Living with HIV
WHO: World Health Organization
