# Supplementary material for: Point-of-care HPV testing for cervical cancer screening in Sub-Saharan Africa: platform diversity, diagnostic performance, implementation feasibility, and determinants—a scoping review with contextual considerations for Ethiopia
Source: BMC Public Health. 2026 Jan 26;26:990. doi: 10.1186/s12889-026-26382-9 (PMC13020338; doi:10.1186/s12889-026-26382-9)
Supplement: Supplementary file 1 — Supplementary Material 1. [file 12889_2026_26382_MOESM1_ESM.docx]

**Supplementary Table S1: Point-of-Care Testing Platforms, Study Designs, and Participant Characteristics in Sub-Saharan Africa**

| **Author(years)** | **Country** | **POC platform** | **Study setting** | **Study design** | **Population** | **Age (years)** | **Sample size** | **Platform utilized** | **Uptake** | **HIV status** | **Specimen type** | **Collected by** |
| --- | --- | --- | --- | --- | --- | --- | --- | --- | --- | --- | --- | --- |
| **Carla J. Chibwesha et al. (2016)** | Zambia | Xpert HPV | Clinic | Cross-sectional | HIV-infected women ≥18, non-pregnant | Median 42 ( IQR 34–47) | 200 | 100% | 100% | All HIV-infected | Cervical | Provider |
| **Carla J. Chibwesha et al. (2016)** | Zambia | OncoE6^TM^ | Clinic | Cross-sectional | HIV-infected women ≥18, non-pregnant | Median 42 (IQR 34–47) | 200 | 100% | 100% | All HIV-infected | Cervical | Provider |
| **Mwenda V et al. (2023)** | Kenya | Xpert HPV | Community | Pilot implementation | Community-screened women 30–49 | 15.6% <30, 52.8% 30–49, 9.7% ≥50 | 4500 | 27% of target | 7% | NR | Cervical | Self & Provider |
| **Maina T et al. (2020)** | Kenya | careHPV | Clinic | Cross-sectional | Women 20–48 attending routine screening | 20–48 (Mean 35) | 283 | 63.7% | 63.7% (actual 86.6%) | HIV-uninfected: 93, HIV-infected: 62 | Cervical | Provider |
| **Saidu et al. (2021)** | South Africa | careHPV | Clinic/Outreach | Prospective observational | Women 30–65, HIV+ and HIV− | Median 39–42 | Screening: 705, Referral: 402 | 100% | Self: 61.5% HIV+, 25.1% HIV−; Clinician: 48.2% HIV+, 16.3% HIV− | 50% HIV+ | Vaginal & Cervical | Self & Provider |
| **Taghavi K et al. (2024)** | Zambia | Xpert HPV | Clinic | Paired prospective | WLHIV 18–65 | Median 37 (IQR 31–44) | 375 | 100% | 100% | All WLHIV | Cervical | Provider |
| **Segondy et al. (2016)** | Burkina Faso & South Africa | careHPV | Clinic | Cohort | Women 25–50, HIV+ | 25–50 | 1052 | 100% | 84.3% | HIV+ | Cervical | Provider |
| **Dorcas Obiri-Yeboah et al. (2017)** | Ghana | careHPV | Clinic | Comparative | Women ≥18, HIV+ and HIV− | 43–45 | 175 | 50% | 89% | HIV+ 94, HIV− 81 | Cervical | Provider |
| **Ngou et al. (2013)** | South Africa & Burkina Faso | careHPV | Clinic | Cross-sectional | HIV-1 infected women 25–50 | 25–50 | 149 | 100% | 100% | HIV+ | Cervical | Cervical |
| **Naomi et al. (2020)** | Tanzania | AmpFire | Clinic | Cross-sectional | Pregnant women >8 weeks gestation | NR | 385 | 100% | 100% | NR | Vaginal | Provider |
| **Murangwa et al. (2022)** | Rwanda | Xpert + AmpFire | Clinic | Cross-sectional | WLHIV | Adult women | 298 | 95% | 47.3% | HIV+ | Cervical | Provider |
| **Mremi et al. (2022** | Tanzania | careHPV | Clinic | Cross-sectional & cohort | Women 25–60 | 25–60 | 1620 | 31.6% | 96.9% follow-up | Any women | Cervical | \| Self \| \| --- \| |
| **Sikhulile Moyo et al. (2023)** | Botswana | AmpFire vs Xpert | Laboratory | Validation study | WLHIV | NR | 63 | 11.1% (7 stored cervical specimens) | NR | HIV+ | Cervical | Provider |
| **Mbulawa et al. (2017)** | South Africa | Xpert HPV | Clinic | Observational | HIV-infected women | NR | 1161 | 100% | NR | HIV+ | Cervical | Provider |
| **Effah K et al. (2023** | Ghana | careHPV | Clinic | Retrospective | Women presenting for screening | 39.2 ± 9.4 | 1414 | 23.3% | NR | NR | Cervical | Provider |
| **Effah K et al. (2023)** | Ghana | AmpFire HPV | Clinic | Retrospective | Women presenting for screening | 39.6 ± 9.2 | 3377 | 55.8% | NR | NR | Cervical | Cervical |
| **Desai et al. (2022)** | Nigeria | ScreenFire | Community | Cross-sectional | Women attending screening | 16–88 (mean 44) | 453 | 100% | NR | NR | Cervical | Provider |
| **Luckett R et al. (2025)** | Botswana | AmpFire HPV | Clinic | Prospective cohort | Women ≥25, HIV+ and HIV− | Mean 42 ± 11 | 2957 | 100% | NR | HIV+ 1479, HIV− 1478 | Vaginal & Cervical | Self & Provider |
| **Kuhn et al. (2020)** | South Africa | Xpert HPV | Clinic | Diagnostic accuracy | Women 30–65, HIV+ and HIV− | 30–65 | 1121 | NR | NR | HIV+ 535, HIV− 586 | Cervical | Provider |
| **Louise Kuhn et al. (2017)** | South Africa | Xpert HPV | Clinic | Cross-sectional | Women 30–60, HIV+ and HIV− | Mean 41–44 | 529 | NR | NR | HIV+ 250, HIV− 279 | Cervical & Vaginal | Provider & Self |
| **Maria J. Barra et al. (2025)** | Mozambique | LAMP assay | Clinic | Analytical evaluation | Women, cervicovaginal swabs | 30–49 | 191 | 100% | Not applicable | Not stratified | Cervical & Vaginal | Self & Provider |
| **Mungo et al. (2024)** | Malawi | ScreenFire vs Xpert | Laboratory | Analytical comparison | WLWH | 25–50 | 315 | Xpert 70%, ScreenFire 75–90% | NR | All WLWH | Cervical & Vaginal | Self & Provider |
| **Johnson Katanga et al. (2019)** | Tanzania | careHPV | Clinic | Cross-sectional | Women 25–60 | 25–60 (mean 40) | 3640 | 100% | NR | HIV+ & HIV− | Cervical | Provider |
| **Cholli P. et al. (2017)** | Cameroon | careHPV | Clinic | Prospective | Women ≥30, HIV+ and HIV− | ≥30 | 913 | 24% | 56% follow-up | 42% HIV+ | Cervical | Provider |
| **Johnson Katanga et al. (2021)** | Tanzania | careHPV | Clinic | Comparative diagnostic | Women routine screening | 25–60 | 3643 | NR | NR | Reported as subgroups | Cervical | Provider |
| **Cubie H et al. (2017)** | Malawi | Xpert HPV | Clinic | Descriptive | Women eligible for screening | NR | NR | NR | NR | NR | Cervical | Provider |
| **Denny et al. (2023)** | South Africa | Xpert HPV | Clinic | Demonstration study | Women 30–65, HIV+ and HIV− | 30–65 | \|  \| \| --- \|  \| 3062 \| \| --- \| | 100% | 94.6% | HIV+ 44%, HIV− 56% | Cervical | Provider |
| **Jose Jeronimo et al. (2014)** | Uganda | careHPV | Clinic | Cross-sectional | Women eligible for screening | 25–60 | 16,951 | 100% | 100% | NR | Cervical & Vaginal | Provider & Self |
| **Fitzpatrick MB (2019)** | Zimbabwe | Xpert HPV | Community | Cross-sectional | Women rural communities | Mean 43.8 | 643 | 100% | 70% self-collection | HIV+ 123, HIV− 520 | Vaginal | Self |
| **Esber et al. (2018)** | Malawi | GeneXpert HPV | Clinic | Cross-sectional | Care-seeking, unscreened women | 18–49 (median 32–34) | 199 | ~100% | ~100% | ~100% | Cervical & Vaginal | Self & Provider |
| **Elliott T. et al. (2019)** | Botswana | Xpert HPV | Clinic | Cross-sectional | WLHIV ≥25 | Median 44 | 103 | 100% | 100% | HIV+ | Vaginal & Cervical | Self & Provider |
| **Downham et al. (2024)** | Senegal, South Africa | OncoE6/E7 (8-HPV) | Laboratory | Randomized controlled trial | HPV+ women, HIV+ & HIV− | 25–54 | 303 | 100% | 100% | HIV− & WLWH | Cervical | Provider |
| **Abate et al. (2025)** | Ethiopia | OncoE6™ | Clinic | Cross-sectional | Women >30 or HIV+ | Median 49 | 297 | 100% | 100% | HIV+ 59, HIV− 238 | Cervical | Provider |

**IQR=Interquartile range; NR=Not reported; WLWH/WLWHIV= Women living with HIV**
