# Supplementary material for: Point-of-care HPV testing for cervical cancer screening in Sub-Saharan Africa: platform diversity, diagnostic performance, implementation feasibility, and determinants—a scoping review with contextual considerations for Ethiopia
Source: BMC Public Health. 2026 Jan 26;26:990. doi: 10.1186/s12889-026-26382-9 (PMC13020338; doi:10.1186/s12889-026-26382-9)
Supplement: Supplementary file 2 — Supplementary Material 2. [file 12889_2026_26382_MOESM2_ESM.docx]

**Supplementary Table S2: Diagnostic Accuracy of Point-of-care HPV Tests Across Studies in Sub-Saharan Africa**

| **Author (years)** | **Country** | **POC platform** | **HPV positivity (%)** | **High-risk HPV types detected** | **Sensitivity (%)** | **Specificity (%)** | **PPV (%)** | **NPV (%)** | **Reference standard** |
| --- | --- | --- | --- | --- | --- | --- | --- | --- | --- |
| **Carla J. Chibwesha et al. (2016)** | Zambia | Xpert HPV | 63% | HPV16; HPV18/45; pooled 11 types (31/33/35/39/51/52/56/58/59/66/68) | CIN2+ 88% (71–97); CIN3+ 90% (68–99) | CIN2+ 60% (52–68); CIN3+ 57% (48–65) | 19% (12–29) | 98% (93–100) | Histology (CIN2+/CIN3+) |
| **Carla J. Chibwesha et al. (2016)** | Zambia | OncoE6^TM^ | 6% positive; HPV16: 5%; HPV18: 2% | HPV16, HPV18 E6 oncoprotein | CIN2+ 31% (16–50%); CIN3+ 40% (19–64) | CIN2+ 99% (97–100%); CIN3+ 98% (95–100%) | 73% (39–94) | 94% (89–97) | Histology (CIN2+/CIN3+) |
| **Taghavi K, et al. (2024)** | Zambia | Xpert HPV | 43.5% | HPV16, HPV18/45, other hrHPVs | 67.3% (57.7–75.7) | 65.3% (59.4–70.7) | 36.4% | 79.1% | Histopathology (biopsies baseline + 6 months) |
| **Segondy et al. (2016)** | Burkina Faso & South Africa | careHPV | 45.1% | HPV16,18,31,33,35,39,45,51,52,56,58,59,66,68 | Burkina Faso 100% (66.4–100), South Africa 92.2% (81.1–97.8) | Burkina Faso 54.7% (49.9–59.5), South Africa 60.9% (56.3–65.5) | Burkina Faso 4.4% (2–8.1), South Africa 21.2% (16–27.1) | Burkina Faso 4.4% (2–8.1), South Africa 21.2% (16–27.1) | Histology (CIN2+) or HSIL |
| **Dorcas Obiri-Yeboah et al. (2017)** | Ghana | careHPV | \|  \| \| --- \|  \| 55% overall; HIV+:79%; HIV–:28% \| \| --- \| | 14 hr-HPV types | HIV+: 97.3%; HIV–: 95.7% | HIV+: 85%; HIV–: 93.1% | HIV+: 96%; HIV–: 84.6% | HIV+: 89.5%; HIV–: 98.2% | Anyplex II HPV 28 assay |
| **Abate et al. (2025)** | Ethiopia | OncoE6™ Cervical Test | 12.46% | HPV16, HPV18 E6 oncoprotein | 57.14% (43.22–70.29) | 98% (95.23–99.32) | 86.6% (71.36–95.53) | 90.76% (86.49–93.93) | Histopathology (punch biopsy) |
