# Supplementary material for: Point-of-care HPV testing for cervical cancer screening in Sub-Saharan Africa: platform diversity, diagnostic performance, implementation feasibility, and determinants—a scoping review with contextual considerations for Ethiopia
Source: BMC Public Health. 2026 Jan 26;26:990. doi: 10.1186/s12889-026-26382-9 (PMC13020338; doi:10.1186/s12889-026-26382-9)
Supplement: Supplementary file 3 — Supplementary Material 3. [file 12889_2026_26382_MOESM3_ESM.docx]

**Supplementary Table 3: Implementation Models, Acceptability, Feasibility, Barriers, Facilitators, and Sustainability of Point-of-care HPV Tests in Sub-Saharan Africa**

| **Author (years)** | **Country** | **POC platform** | **Implementation model** | **Women acceptability** | **Provider acceptability** | **Feasibility** | **Barriers** | **Facilitators/implementation factors** | **Sustainability** | **Conclusion** |
| --- | --- | --- | --- | --- | --- | --- | --- | --- | --- | --- |
| **Carla J. Chibwesha et al. (2016)** | Zambia | Xpert HPV | Validation-only | Cost acceptable; POC available | Minimal training required | Compatible with HIV/TB lab systems | Regulatory constraints; cartridge costs | Existing GeneXpert network | High | High sensitivity; suitable for LMIC screen-and-treat |
| **Carla J. Chibwesha et al. (2016)** | Zambia | OncoE6^TM^ | Validation-only | Acceptable; inexpensive | NR | Simple collection | Regulatory constraints | NR | Limited | High specificity; low sensitivity; not stand-alone for primary screening |
| **Mwenda V et al. (2023)** | Kenya | Xpert HPV | Community outreach + same-day VIA triage | High; Self-sampling preferred; fast results | Workflow challenge | Integrates with HIV/TB clinics | Low triage compliance; staff turnover; stock-outs | Community engagement; self-sampling, training | Requires strengthening | Feasible but needs throught and follow-up systems |
| **Maina T et al. (2020)** | Kenya | CareHPV | Potential POC (not same-day tested) | Low-cost; acceptable | Simple workflow; suitable for LMIC clinics | Minimal equipment requirement | Costs; training gaps; stockouts | VIA integration | NR | Promising but limited by ow sensitivity |
| **Saidu et al. (2021)** | South Africa | Xpert HPV | same-day (~1 h); Screen-and-treat | High; self-samplingpreferred | high; simple workflow | Minimal electricity; portable device | Low specificity among WLHIV; quality assurance; cost | Self-sampling; WHO-prequalified | Scalable via GeneXpert | Feasible; improves coverage |
| **Taghavi K et al. (2024)** | Zambia | Xpert HPV | Same-day POC at HIV clinic | High | Acceptable with nurse-led | Integrated easily; 2–4 h result | Misses some CIN2+; limited follow-up | Strong workflow and training | High due to GeneXpert widely | Scalable with quality assurance |
| **Segondy et al. (2016)** | Burkina Faso, South Africa | careHPV | Same-day screen-and-treat | High; fast results | Affordable; practical | Works in low-resource settings | Low specificity | Donation-supported kits; training | Supported via donation programs | Proven feasibility; affordable |
| **Dorcas Obiri-Yeboah et al. (2017)** | Ghana | careHPV | Same-day screen and treat (~2.5–3 h) | High self-collection possible | Minimal technical skill required | Portable; battery backup | Reliance on donated kits | Simple workflow | Scalable | Suitable for expanding HPV-based screening |
| **Ngou et al. (2013)** | South Africa, Burkina Faso | careHPV | Same-day screen | High | Performed by trained scientists | Minimal laboratory requirements | Cross-reactivity | Affordable | NR | Feasible for large-scale screening |
| **Naomi et al. (2020)** | Tanzania | AmpFire HPV | Antenatal clinic-based | High;  Self-collection | NR | Isothermal; rapid | NR | Fits ANC workflows; minimal tools/training | Promising | Requires validation |
| **Murangwa et al. (2022)** | Rwanda | Xpert HPV + AmpFire | NR | high | NR | Low-cost; fast (~1 h) result | Follow-up delays | Integration with HIV care | Supports scale-up | Good agreement; feasible |
| **Mremi et al. (2022)** | Tanzania | careHPV | NR | High; self-sampling | Nurses supported self-sampling | Easy in rural primary health centers | Limited awareness | Free screening; SMS-reminders | NR | Feasible ;increases rural coverage |
| **Sikhulile Moyo et al. (2023)** | Botswana | AmpFire vs Xpert HPV | Potential same-day test-and-treat | High; self-sampling | Easy to use | Minimal extraction; compatible low-resource | Lab infrastructure limitations | Affordable; provides genotyping | NR | Suitable for HIV+ women Excellent performance |
| **Mbulawa et al. (2017)** | South Africa | Xpert HPV | Lab-based | NR | NR | Automated; ˜1hr | Cartridge cost | Rapid; automated | High | Useful for WLWH |
| **Effah K et al. (2023** | Ghana | careHPV | Batch processing | High | Moderate | Low-power; low-cost | Batch delays | Simple workflow battery-operated | Good for small programs | Effective in low-resource settings |
| **Effah K et al. (2023)** | Ghana | GeneXpert HPV | Same-day (~1 h) | Moderate | Limited by Low throughput | Stable power required | Low throughput; cost to patients | Integration with TB program | Limited | Useful but limited by cost |
| **Desai et al. (2022)** | Nigeria | ScreenFire | Screen-and-treat; risk-based triage | High self-compatible | Easy workflow | No DNA extraction | Reagent competition | Low cost; risk- stratification | Multisite potential/deployment | Accurate; low-cost |
| **Luckett R et al. (2025)** | Botswana | AmpFire HPV | Same-day triage | high | Feasible; reduced workload | Low electricity needs | Standard LMIC constraints | Self-sampling; Ct-value for triage | Feasible | Reduces overtreatment |
| **Kuhn et al. (2020)** | South Africa | Xpert HPV | Screen-and-treat | High | Acceptable | Low-resource compatible | Standard (resource) barriers | Adjustable assay cutoffs | Scalable | Improved specificity with modified cut-offs |
| **Louise Kuhn et al. (2017)** | South Africa | Xpert HPV | NR | NR | NR | NR | NR | Self-collection feasible; partial genotyping | NR | Useful for triage optimization |
| **Maria J. Barra et al. (2025** | Mozambique | LAMP (DARQ) | Same-day | High; <1 h | NR | Portable; Minimal electricity | Extraction-free sensitivity concerns | Low-cost; simple workflow; internal control | Promising | Sensitive; low-cost POC |
| **Mungo et al. (2024)** | Malawi | ScreenFire vs Xpert HPV | Laboratory-only | Not measured | Not measured | Isothermal;  batch | Xpert cost | High concordance | NR | Low-cost alternative; Good CIN2+ detection |
| **Johnson Katanga et al. (2019)** | Tanzania | careHPV | Delayed processing | High; self-collection | Moderate | Low electricity | Stock-outs; training gaps; cartridge cost | Local lab capacity | High  Linkage (HIV+) | Highly sensitive; triage improves specificity |
| **Johnson Katanga et al. (2021)** | Tanzania | careHPV | NR | NR | NR | Processed at clinics | NR | NR | NR | Good agreement with HC2 |
| **Cholli P. et al. (2017)** | Cameroon | careHPV | Same-day treatment | NR | NR | Clinic-based processing | NR | NR | NR | Co-testing reduces overtreatment |
| **Cubie H et al. (2017)** | Malawi | Xpert HPV | NR | High | Feasible with minimal manual steps | Small footprint | Stock-outs, cartridge cost, cold chain | VIA integration | NR | Supports same-day screen-and-treat |
| **Denny et al. (2023)** | South Africa | Xpert HPV | Screen & Treat | High | Feasible | Works in primary care | NR | Same-day results enable treatments | High | Improves linkage and prevention |
| **Jose Jeronimo et al. (2014)** | Uganda | careHPV | Same-day | High; self-sampling | Feasible | Low power; moderate equipment | Stock-outs; training gaps | Self-collection | Good follow-up | Feasible; acceptable |
| **Fitzpatrick MB (2019)** | Zimbabwe | GeneXpert HPV | Near POC; outreach | High | Feasible with used community health workers | Minimal electricity | Power; sample transport | Integration with ART; community outreach | NR | Suitable for screening |
| **Esber et al. (2018)** | Malawi | GeneXpert HPV | Clinic based; same-day | Very  High (>95%) | high | Easy; minimal power | Transport medium cost | Existing GeneXpert network | VIA referral | Feasible in rural clinics |
| **Elliott T. et al. (2019)** | Botswana | GeneXpert HPV | Pilot; near POC | High; self-sampling | Workflow feasible | Rapid ~60 min | Shared machine | Integration; rapid results | Moderate | Accurate; supports scale-up |
| **Downham et al. (2024)** | Senegal, South Africa | OncoE6/E7 (8-HPV) | Screen-triage-treat | 70% suitable | >80% easy; ~90% required moderate lab experience | 4 h run; batch | Time-consuming | Suitable for low-resource settings | NR | Operationally  feasible; needs refinement |
| **Abate et al. (2025)** | Ethiopia | OncoE6^TM^ | Facility-based;2.5 h; not same-day treatment | NR | NR | Lateral-flow POC; simple | Limited genotype coverage | High specificity; local production | NR | Sub-optimal sensitivity; best combined with other tests |

**VIA=Visual inspection with ascetic acid; TB=Tuberculosis; HC2= Hybrid capture 2; ANC= Antenatal care; SMS=Short messaging service; Ct=cycle threshold; ART=Antiretroviral therapy**
